# Editorial: Sex steroid hormones: effects on breast cancer risk and etiology

**DOI:** 10.3389/fendo.2023.1198770

**Published:** 2023-04-19

**Authors:** Noha A. Mousa, Natascia Marino, Bruno M. Simões

**Affiliations:** ^1^ Clinical Sciences Department, College of Medicine, University of Sharjah, Sharjah, United Arab Emirates; ^2^ Department of Medicine, Indiana University School of Medicine, Indianapolis, IN, United States; ^3^ Manchester Breast Centre, Division of Cancer Sciences, School of Medical Sciences, Faculty of Biology, Medicine and Health, University of Manchester, Manchester Academic Health Science Centre, Manchester, United Kingdom

**Keywords:** breast cancer, risk, sex steroid hormones, estrogen, chemoprevention

A key distinctive feature of breast tissue is its dependence on female sex steroid hormones, which are responsible for epithelial cell proliferation and normal development of the mammary gland. In the same way, breast cancer (BC) is frequently dependent on sex hormones for its growth, providing the rationale to reduce their levels or antagonize their action as a key treatment for the great majority of patients affected by this disease. Anti-hormonal therapies have also been used to develop effective BC preventive interventions (1). Hence, the main strategy to reduce BC risk relies on lowering the exposure of the breast glandular tissue to the female sex steroid hormones; specifically, estrogen and progesterone. Several “sex-hormonal-modulating” lifestyle, medical, and surgical interventions have demonstrated evident efficacy in reducing BC risk in healthy women who had no prior history of BC. Likewise, lifestyle and exogenous sex steroids have been linked to a significant increase in BC risk. In this topic collection, researchers draw attention to four different aspects highlighting the role of sex steroids in the management of hormonal BC risk.

Whenever BC risk is discussed, the modifiable factors become of crucial practical importance. Extensive research evidence supports the effect of lifestyle factors such as diet, alcohol, and physical activity on BC risk (2). Yet, a limited number of studies focused on their impact on the circulatory levels of sex steroid hormones as a potential predictor of that risk. Wiggs et al. share a well-needed review on the effect of diet and exercise on the levels of different forms of estrogen and their key metabolites. The article discusses recent evidence from large clinical studies endorsing the potential benefit of weight loss, *via* either diet or diet associated with an exercise intervention, for BC prevention *via* modulation of endogenous estrogen levels.

There are recognized epidemiological and clinical evidence that lifetime exposure to high levels of circulatory steroid hormones may increase BC risk. Indeed, some of the validated BC risk models, such as the Gail and Tyrer-Cuzick models, calculate a higher estimated BC risk for women who have prolonged exposure to ovarian hormones as a result of early age at menarche and late age at menopause (3, 4). As well, the exaggerated peripheral tissue synthesis of estrogens due to an overexpression of the aromatase gene is linked to BC risk. In this topic collection, Man et al. looked into an under-studied inherent risk factor relevant to aberrant estrogen production by the ovaries. Using an ovarian cell line model, they studied the effect of overexpressing TOX3, recently identified as a BC susceptibility gene, on estrogen synthesis. Their *in vitro* findings suggest that aberrant expression of estrogen biosynthesis-mediating genes, like TOX3, may result in the exposure of women to higher-than-normal levels of circulatory estrogens. As such, the study proposes increased BC risk as a consequence of estrogen overproduction which may lead to abnormal activation of the estrogen signaling pathway in the breast epithelial cells.

In addition to the endogenous hormones, exogenous sex steroids are widely used by women for short and long-term family planning. A 2019 United Nations report estimated that 248 million women were using hormonal contraceptives. These include different forms of synthetic estrogen, progesterone, or a combination with diverse pharmacologic characteristics, and thus physiologic or potential pathologic effects. Recently, several large-scale clinical studies provided evidence about the BC risk associated with the use of each contraceptive method. However, one of the newer generation methods, the progestogen subdermal implant, has received less attention in terms of its association with BC risk. With a striking rise in its use by more than 20 times over the past two decades, Mohammed et al. raise the attention of clinicians and researchers on a potential associated BC risk that is not yet well presented in current clinical guidelines. They highlight the need of addressing such risk during counseling women contemplating this method of contraception, especially those already at high BC risk because of hereditary or acquired risk factors (i.e., reproductive factors and obesity). Interestingly, more recent studies have confirmed the significant association of BC risk with the use of progestogen-only contraceptives, including the subdermal implant (5, 6).

Several multi-center placebo-controlled randomized clinical trials gathered consistent evidence for the effectiveness of chemopreventive therapies in reducing BC risk in high-risk women or the general population [reviewed in (2)]. Selective estrogen receptor modulators (SERMs), mainly Tamoxifen and Raloxifene, were reported to achieve significant BC risk reduction of approximately 38%. Nevertheless, the associated serious adverse events of endometrial cancer and thromboembolism led to a considerable limitation on their use for prevention. Aromatase inhibitors (AIs) have shown superior effectiveness in BC therapy compared to tamoxifen and have also been investigated for their effectiveness in reducing BC risk in high-risk women. Two key clinical trials (MAP.3 and IBIS-II) showed a significant 53-54% reduction of BC incidence in women who used AIs (1). The superior effectiveness is mainly due to the induction of a state of near-total estrogen deprivation, in contrast to SERMs which solely act as receptor antagonists. However, despite the overall safety of AIs, such potency comes at a cost. Hyder et al. discuss, in an exhaustive well-structured review, an important barrier preventing the wide application of AIs in BC chemoprevention. The “Aromatase Inhibitor-Associated Musculoskeletal Syndrome” is the main reason for the premature discontinuation of AIs by patients. The authors discuss its mechanism, risk factors, and evidence about available management options, stressing the need to develop effective therapeutic strategies for AI-related adverse effects.

In summary, this Research Topic illustrates the extent and impact of sex steroid hormones in the etiology and risk of BC development and contributes to answering the key Research Topic question of “which environmental and genetic factors regulate hormone levels among pre-and post-menopausal women?”. The articles provide a better knowledge of the mechanisms involved in the pathogenesis of BC that could lead to improved patient stratification and preventive interventions.

## Author contributions

All authors listed have made a substantial, direct, and intellectual contribution to the work and approved it for publication.
